# HIV HGM biobank as a research platform for paediatric infectious diseases and COVID-19 pandemic

**DOI:** 10.1186/s12981-022-00448-1

**Published:** 2022-05-25

**Authors:** Consuegra Irene, Mauleón Elba, José Luis Jiménez, María José Mellado, María Ángeles Muñoz-Fernández

**Affiliations:** 1 Instituto de Investigación Sanitaria Gregorio Marañón (IiSGM), Madrid, Spain; 2 HIV HGM BioBank, Madrid, Spain; 3 Plataforma-Laboratorio (IiSGM), Madrid, Spain; 4 General Pediatrics, Infectious and Tropical Diseases Department Hospital, Universitario La Paz, Madrid, Spain; 5 Laboratorio InmunoBiología Molecular (HGUGM), Madrid, Spain; 6 Head Immunology Section, Hospital General Universitario Gregorio Marañón, C/Dr. Esquerdo 46, 28007 Madrid, Spain

**Keywords:** BioBank, HIV, COVID-19, Paediatric, Pandemics, Research infrastructure

## Abstract

**Aim:**

The initial cases of COVID-19 appeared in December 2019 and Spain was one of the most affected countries during the first wave (March to June). Since then, HIV HGM BioBank has been restructured as an established Paediatrics and Adults HIV_COVID-19 BioBank that aims at the long-term storage of samples obtained from not only HIV-1, but also from COVID-19 patients and HIV-1_COVID-19 coinfected patients.

**Methods:**

HIV HGM BioBank holds high quality biological samples from newborns, children, adolescents and adults with their associated clinical data. Research groups trying to establish large networks focused on research on specific clinical problems in epidemiology, biology, routes of transmission and therapies, are potential users of the clinical samples and of associated data of HIV-1_COVID-19 HGM BioBank.

**Results:**

The HIV HGM BioBank is an academic and ethical enterprise complying with all the legal regulatory rules to provide service to the society. HIV_COVID-19 HGM BioBank has been repurposed to offer an important resource for global research of COVID-19 in newborns, children, adolescents, adults and elders to study the biological effect of the pandemic.

**Conclusion:**

Herein, we present a description of how HIV HGM BioBank has rapidly become an indispensable structure in modern biomedical research, including COVID-19 research.

## Keypoints

Biobanks are essential platforms for the development of personalized medicine, even more so in pediatric diseases, including those of infectious origin. The possibility of having collections of excellent quality and with all the ethical and legal requirements is even more relevant in pandemic situations.

## Introduction

In the year 2020, a new infectious respiratory disease emerged in Wuhan, Hubei province, China [1, 2]. An initial cluster of infections was linked to Huanan seafood market, potentially due to a human contact with animal species. Subsequently, human-to-human transmission occurred [3] and the disease now termed, coronavirus disease 19 (COVID-19) rapidly spread within China [1, 4]. The first cases of COVID-19 occurred in December 2019 and Spain was one of the most affected countries during the first wave of COVID-19 (March to June) [5–11]. Then it was devastated by a second wave of COVID-19 infections [12, 13] and all subsequent waves that have devastated the entire planet and caused the greatest medical problem experienced by humanity in recent decades. In the severe pandemic context biobanks have become even more essential than before [14–17]. They are professional repositories of biological samples. In Spain, in 2004, the Hospital General Universitario Gregorio Marañón founded a national biobank, named HIV HGM BioBank, with the aim of contributing to advance understanding of different pathologies through the transfer, management, register, processing, cryopreservation and cession of biological material from patients, always for research purposes and under conditions that guarantee its usefulness in current studies and future research that may appear as knowledge evolves. This biobank is located in Hospital General Universitario Gregorio Marañón, Madrid, one of top 5 hospitals in Spain, and contains a large number of samples and represents a centralized approach to collecting, processing, and storing samples from all over the country. Along its 17 years of trajectory, this platform has focused its excellence on infectious diseases and, more specifically, on paediatric donors. Due to its experience, HIV HGM BioBank is now an international model of management in studies and clinical trials based on cohorts of infant samples. This HIV HGM BioBank holds whole blood and serum, plasma, urine, cerebrospinal fluid, feces and meconium, breastmilk, nasal aspirate, and different types of immune cells and DNA, all stored for research use [18]. Our HIV HGM BioBank is engaged in the physical placement of samples and the full amount of work associated with these samples, and responsible for ethically and accurately data management, related to consent, privacy, and control. In this sense, in Spain, Royal Decree 1716/2011 of November 18, states the basic requirements on authorization and performance of biobanks for biomedical research and the management of human biological samples and their associated data in order to achieve excellence in quality and data integrity. According to this rule, biobanks shall: (i) guarantee the informed consent process; (ii) safeguard the right of the withdrawal of donors’ decisions; (iii) guarantee the right to privacy and respect for the will of the subjects participating in the studies; and (iv) assure the equal distribution of samples. [19, 20]. This biobank represents a novel approach, not only to HIV-1 infection, but also to a COVID-19 research, and specializes in paediatric population [21–25]. This is why the HIV HGM BioBank is of general interest to basic and clinical research teams working on HIV-1 and COVID-19, and also to those groups who try to establish large networks focused on research on specific clinical problems in epidemiology, biology, routes of transmission and potential therapies [26]. It is important to note that biobanks are biomedical, scientific, infrastructural development, and they represent a political, legal, ethical enterprise, being integrated by the regulation, medicine, law and society [27, 28]. The purpose of this Spanish BioBank is to set up the unified findings, and discussions on the design and the selection of equipment, the management development methods and staff training, on the standardization of methods for the collection, processing, shipping and storage of biomaterial of different origins as well as on methods and validations for quality control, creation and use of databases of information accompanying biospecimens. Biobanks have been crucial in the run towards a COVID-19 vaccine and/or treatment and a source of knowledge to understand the possible mechanisms that contribute to the appearance and spread of the most critical pandemic of the last decades [28]. The personnel of Spanish HIV HGM BioBank are responsible for the reception, preservation and storage of multiple samples areas, with a high expertise in infectious diseases and excellence in working with infant samples [28–30]. Taking into consideration the present pandemic context, the number of staff working in the Spanish HIV HGM BioBank has been increased. This increase is also a reflection of the critical situation that the COVID-19 pandemic has caused for those countries in which infectious diseases already represented a very important challenge in their containment and treatment, and serves as a potent reminder of the necessity to reinforce medical and public health capacities. Also the threat of future outbreaks should not be underestimated [31–37].

## Materials and methods

At this moment eighty-five hospitals spread across Spain use the HIV HGM BioBank, which have collections of human biomaterials of individual interest. All the HIV-1 samples collected by our HIV HGM BioBank represent the Spanish HIV-1 infection the same as with COVID-19 infection. Our objectives are: (i) the new structure and function of the HIV_COVID-19 HGM BioBank that releases very efficiently samples to different research project, not only in Spain, but also in other countries, (ii) the importance and contribution of a biobank specialized in paediatric research, even more in a pandemic context when a specific approach for this population is needed. Up to now, for more than 15 years of the existence, the HIV HGM BioBank (web site www.hivhgmbiobank.com) has become not only a Spanish HIV HGM BioBank, but also, since February 2020, a Spanish Coronavirus HIV HGM BioBank. The function of this biobank is crucial for the development of new diagnostic and possible therapies for all persons with infectious diseases, such as HIV-1 and COVID-19 infections and HIV-1/COVID-19 coinfections.

Biobank workflow is maintained in a strictly organized manner. This HIV-1 and COVID-19 BioBank applies standard operating procedures (SOPs) for samples required the preservation of viability, structural integrity, functionality and stability. The SOPs ensure correct implementation of essential biobanking components such as samples, associated databases, donors, ethical approvals and informed consents, acquisition, transport, preparation, analysis process faultlessness, proper storage, conditions and terms samples sharing and ensuring the maintenance of the material stored. One of the main goals of this HIV_COVID-19 HGM BioBank is to recognize the researcher’s needs. Therefore, a researcher must complete a sample release application with the aim to receive HIV-1, COVID-19, HIV/COVID-19 or infectious samples of donated materials from patients. Once the received application is approved by the members of the Scientific Committee of the Spanish HIV HGM BioBank, the researcher signs a Material Transfer Agreement (MTA) with the director of the biobank and the coordinator of the cohort. In return the researcher is obliged to send a scientific report with her/his work results every year and to index a reference in materials & methods and acknowledgements sections.

The samples such as blood, serum, plasma, peripheral blood mononuclear cells (PBMC), pellet cells, DNA, RNA, umbilical cord blood, feces, meconium and breast milk are processed and stored in the Spanish HIV_COVID-19 HGM BioBank. It has been recognized that one of the main objectives is to process, store and provide different samples from HIV-1 and/or COVID-19, not only in adults patients, but also in newborns, neonates, infants, children, adolescents and elders to research projects.

It is important to know that COVID-19 guidelines mandate the use of Biosafety Level 2 (BSL-2) rules for laboratories, including the use of protective equipment freezers, the use of Class II Biological Safety Cabinets, and proper disinfection routines. In other words, conditions under which the high Biosafety Level 3 (BSL-3) practices should be followed when working with culture specimens, with different cell lines. Over time, the operation of the HIV_COVID-19 HGM BioBank will become crucial for any research. As expected, the HIV_COVID-19 HGM BioBank has been set up according to a system of quality management based on the rules written in UNE EN-ISO 9001:2015 that covers the full spectrum of the HIV_COVID-19 HGM BioBank´s operations.

This recently updated HIV_COVID-19 HGM BioBank is run by a scientific director and a data manager who are assisted by a Biomedical Research Scientific Committee. An independent Biomedical Research Ethics Committee (CEIm, Hospital General Universitario Gregorio Marañón reviews the agreements made with the different cohorts regarding the patients’ Informed Consent. (https://www.iisgm.com/organizacion/comisiones/comite-de-etica-de-la-investigacion-con-medicamentos-ceim/). The current COVID-19 pandemic, patient sample collection, processing, and analyses are at the forefront of this emergency. COVID-19 presents some unique issues in the biobanking world. The overwhelming scope of this pandemic, with around 2 million cases globally as of mid-April, has assigned an outstanding role to biobanks as they work with samples essential for developing diagnostics and vaccines. The HIV_COVID-19 HGM BioBank consists of well-trained, professional personnel, adequate facilities, equipment, protective measures and biosafety to incorporate novel functions and deal with samples of a new virus associated with the infectious disease such as the COVID-19. This amplified function of the Spanish HIV HGM BioBank was launched at the beginning of the pandemic to obtain and collect samples from newborns, neonates, infants, children, adolescents, adults and elders infected or who had undergone the infection by COVID-19.

The HIV_COVID-19 HGM BioBank has been created to offer different samples donated by patients with the objective to discover new findings in newborns, children, adolescents and adults infected by HIV-1, COVID-19, or HIV-1/COVID-19 [7]. It is worth noting that biobanks have been identified as a biomedical scientific infrastructural development integrated into the preexisting form of regulation, medicine, law, and society. Strict compliance of ethical norms is always guaranteed. The HIV_COVID-19 HGM BioBank offers samples, technical and scientific advances, establishing interesting support to global research on the HIV-1 and COVID-19 infection diseases. In summary, the main function of this HIV_COVID-19 HGM BioBank is to collect and guard human samples strictly connected with their associated data, guaranteeing the highest quality, confidentiality of donors and complying with current ethical and legal regulations. In fact, our Spanish HIV HGM BioBank stores thousands of samples of healthy donors, HIV-1 individuals, COVID-19 individuals, and HIV-1_COVID-19 coinfected individuals.

## Results

Since its creation, HIV HGM BioBank has processed 472,618 aliquots from 54,106 samples of 18,979 donors. The 94.04% of this material comes from patients of infectious diseases. HIV HGM BioBank holds 21,806 aliquots from 5784 samples of 2065 paediatrics donors, of which the 68.47% are of infectious pathologies. Since the moment the pandemic started, our HIV HGM BioBank has received and processed 2901 aliquots from 1407 samples of 593 donors infected with COVID-19 virus. Thus, 27.79% of those material (806 aliquots) belongs to infant patients. This effort positions this platform as a reference in the research of infectious diseases and/or pediatric field. The relevance of counting with HIV HGM BioBank in the scientific community is described along several quality indicators: (a) samples, aliquots & cohorts at researcher’s disposal; (b) collaborations; (c) donated material; and (d) publications. Those elements are exposed as follows:

## Quality indicators of HIV HGM biobank (2004–2020)

### Biomaterial at scientific disposal

**Fig. 1:**
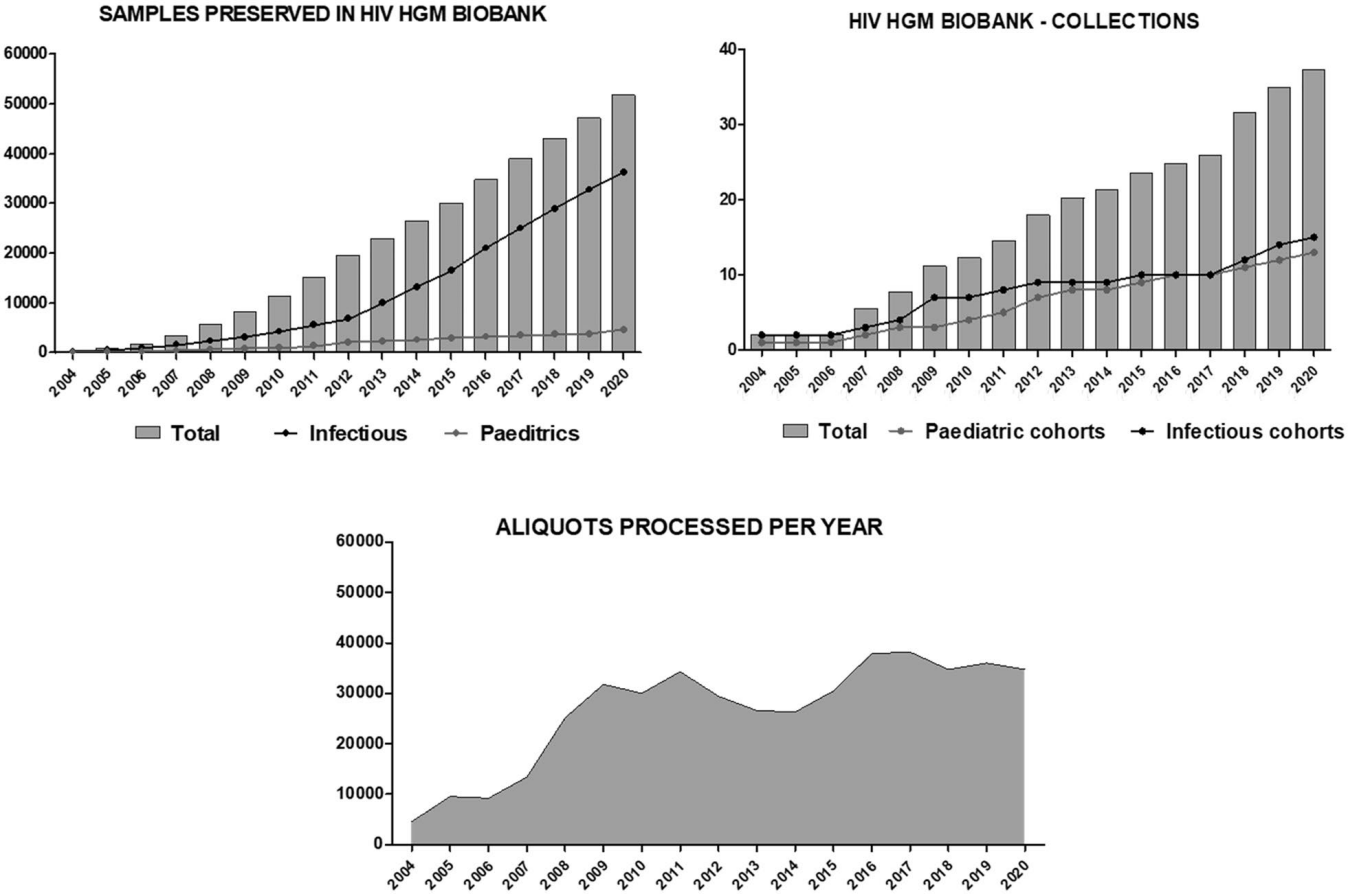
Historical evolution of the material processed and cryopreserved by HIV HGM BioBank

The historical growth ratio for the number of samples preserved in HIV HGM BioBank is + 25% per year, + 10% in the last five years. For the number of aliquots processed each year the ratios are more variable, but HIV HGM BioBank has a + 9% global since 2004, with a record of + 53% and a − 17% minimum. The growth in number of collections managed by HIV HGM BioBank is + 14% per year historically, and + 8% per year for 2016–2020 (Fig. 1).

### Collaborations and services

**Fig. 2:**
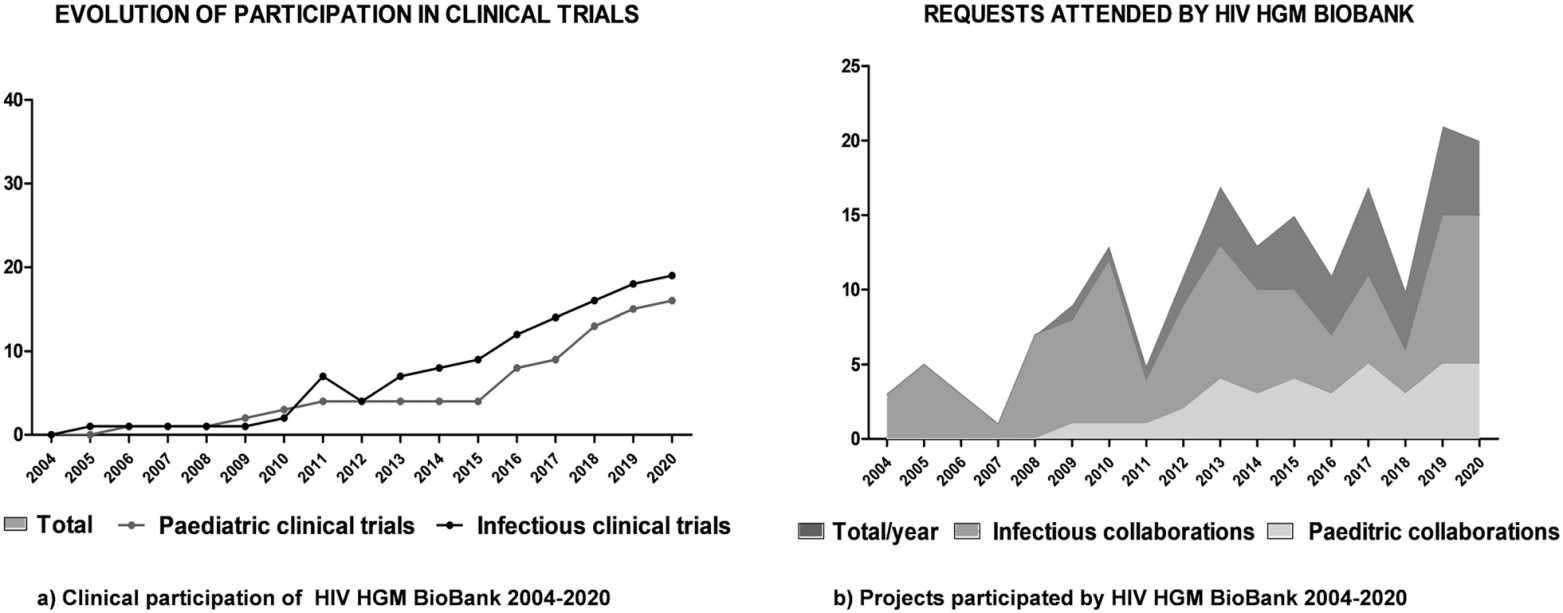
Historical evolution of HIV HGM BioBank participation in research activities

In clinical development the global growth ratio is + 23%, + 17% in the last five years. For research projects with public or private foundation, the growth ratio is + 12% per year (Fig. 2).

### Material donated

**Fig. 3:**
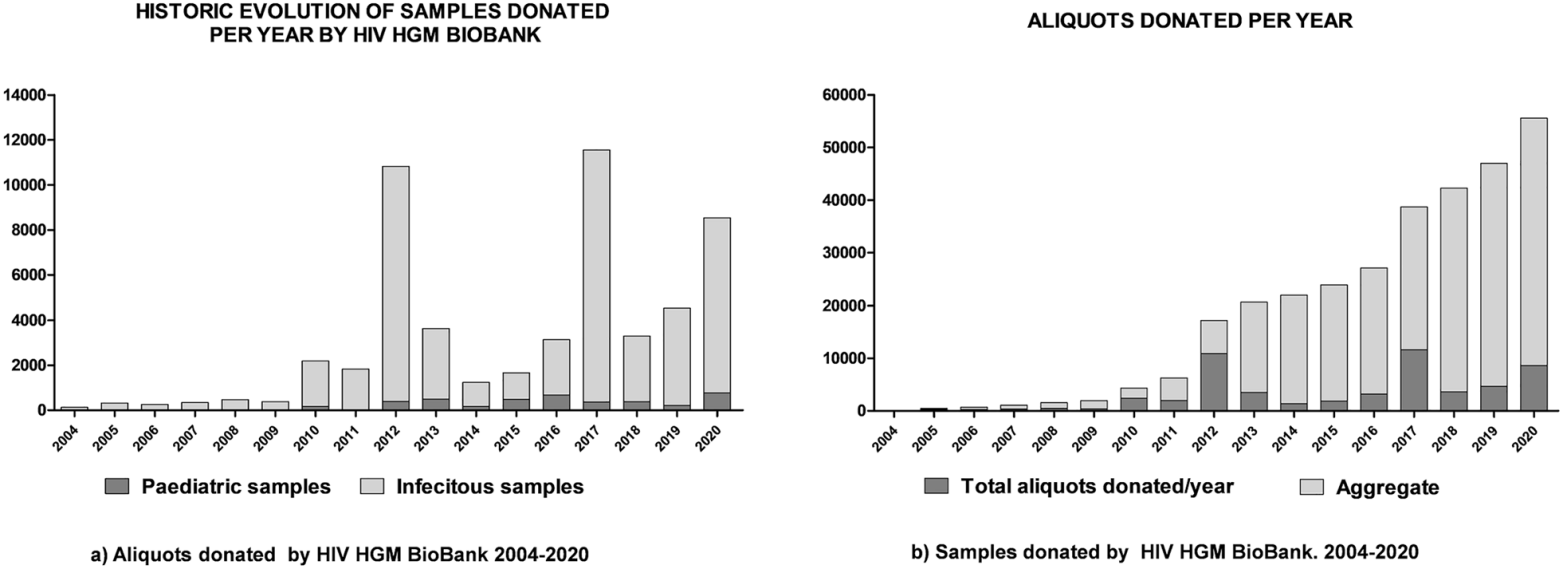
Historical evolution of HIV HGM BioBank participation in research activities by quantity of biomaterial donated

Aliquot donation has developed a global growth ratio of + 28%, + 15% in the last 5 years. The forecasts of samples transfer is one of the main obstacles in biobanking management because it does not suit to the studies of previous demand, so its evolution is more erratic than other indicators. Despite this trait, HIV HGM BioBank has experienced a positive growth since 2004 in sample cession (Fig. 3).

### Results promoted by HIV HGM BioBank

**Fig. 4:**
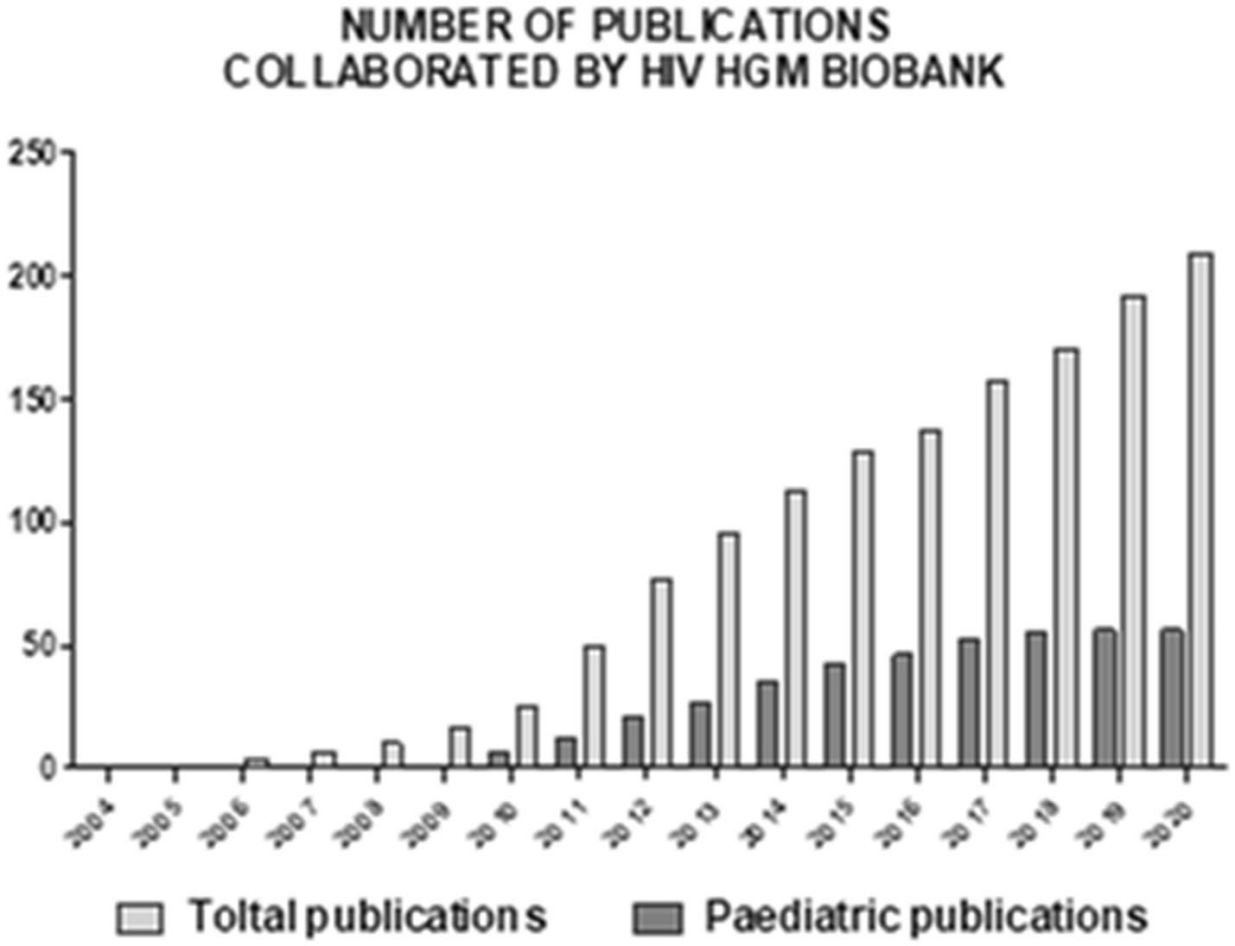
Aggregate of publications with reference to HIV HGM BioBank in “Acknowledgements” and/or “Materials and methods” sections

Papers published thanks to the donation of HIV HGM samples or services, with a global growth ratio of + 27%, + 9% in 2016–2020 period (Fig. 4).

Due to this great expansion in the last five periods, HIV HGM BioBank expects a great development in short time cycles. Also, the specific legislation in biobank activities established in 2012 promotes the use of these facilities in research and clinical trials, improving and driving our requests, services and collaborations. Nowadays, only in the first trimester of 2021, three new clinical trials and 2 paediatric cohorts have been requested.

**Fig. 5:**
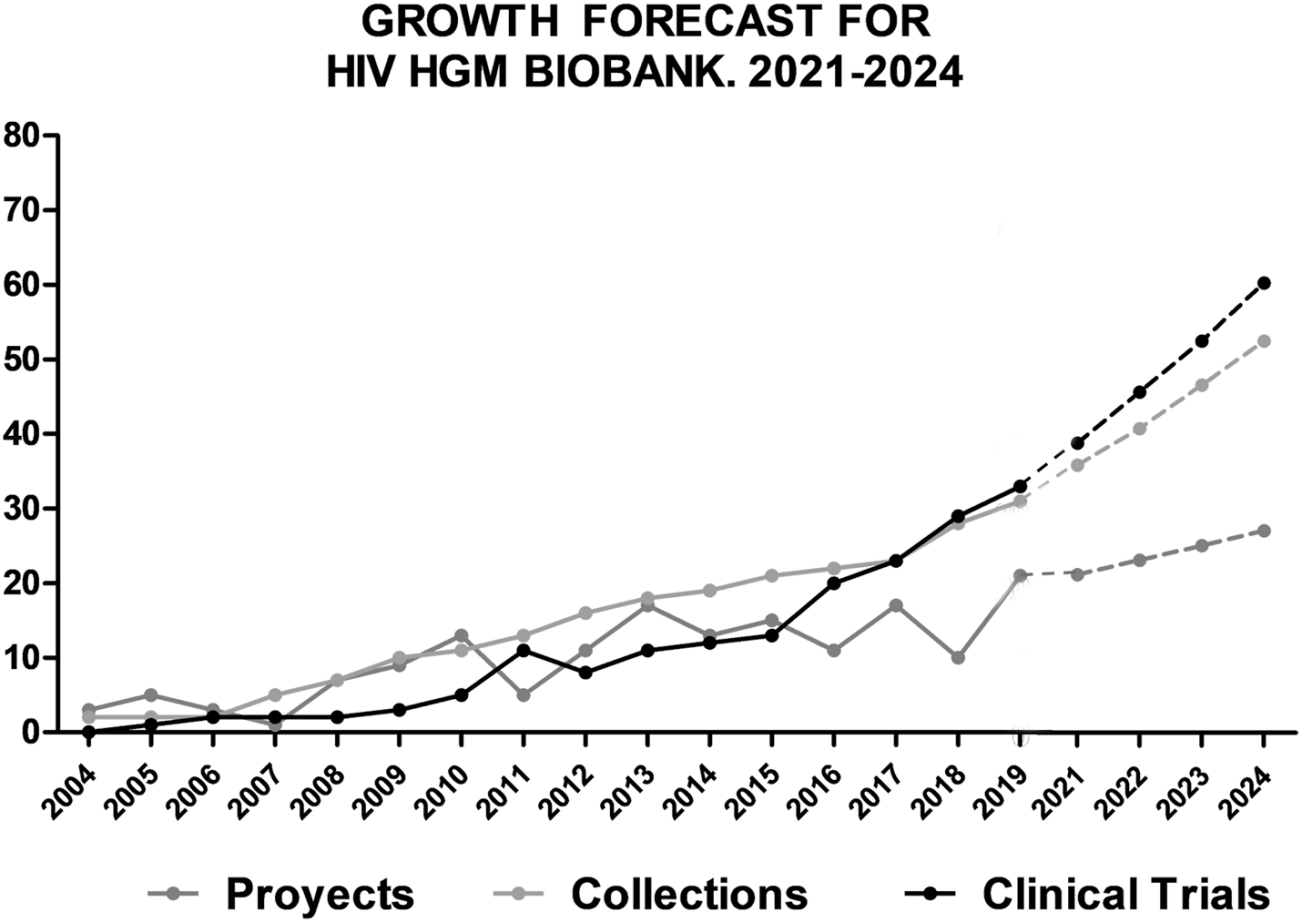
Forecast for short-medium cycle in projects, collections and clinical trials participation of HIV HGM BioBank

The ratios of expected development are + 8% for projects, + 11% collections and + 13% of participation in clinical trials (Fig. 5).

## Conclusion

Biomedical research and the development of personalized medicine go hand in hand with the development of management platforms for large sample collections and big data management, such as biobanks. If these structures were essential before the pandemic, they are even more now that we have discovered that, unfortunately, situations of serious global health crises are unpredictable and unavoidable. It is essential to learn from the experience that COVID-19 has offered us in terms of adapting our structures and knowledge to redirect resources to respond to health problems that society demands to solve urgently. In this sense, it is even more important to have specialized entities, not only in respond to major global challenges, but also in managing the same responses in the pediatric population, whose requirements are specific and sometimes very different from those of the adult population.

In this sense, the HIV HGM Biobank has developed a strategy for rapidly adapting its infrastructures and know-how to the implementation of pediatric and adult collections of COVID19, as well as cohorts of patients co-infected by both pathologies. The possibility of enable to the scientific community pediatric and neonatal samples of excellent quality and a large amount of associated data is especially relevant, since the effects of SARS-COV2 infection in patients with an immature immune system must be studied in depth in the future since its repercussions are still unknown.

In addition, some exceptions were established in the legal requirements for obtaining informed consent, authorized by research ethics committees. A waiver was granted from the need to obtain a signed informed consent during the period of confinement that during the first wave kept the entire population at home. During the first and hardest part of the pandemic, oral consent was valid for the deposit of biological samples in our biobank. This exemption ended in July 2020, and then the donors had to be re-contacted and a written consent formalized. This allowed a more agile and productive management of the deposit of material in the HIV HGM Biobank for these new collections of COVID19 samples. In this way, more material and data were accessed in less time, especially in a moment when achieving critical masses of samples is key to providing tools to the scientific community and being able to provide answers to society. This strategy will allow innumerable studies in the future that will help clarify the mechanisms of action and the possible routes of intervention to eradicate SARS-COV2 in infants and adults.
